# Supplementary figures and images for: Functional analysis of cell lines derived from SMAD3-related Loeys-Dietz syndrome patients provides insights into genotype-phenotype relation
Source: Hum Mol Genet. 2024 Mar 27;33(12):1090–104. doi: 10.1093/hmg/ddae044 (PMC11153339; doi:10.1093/hmg/ddae044)

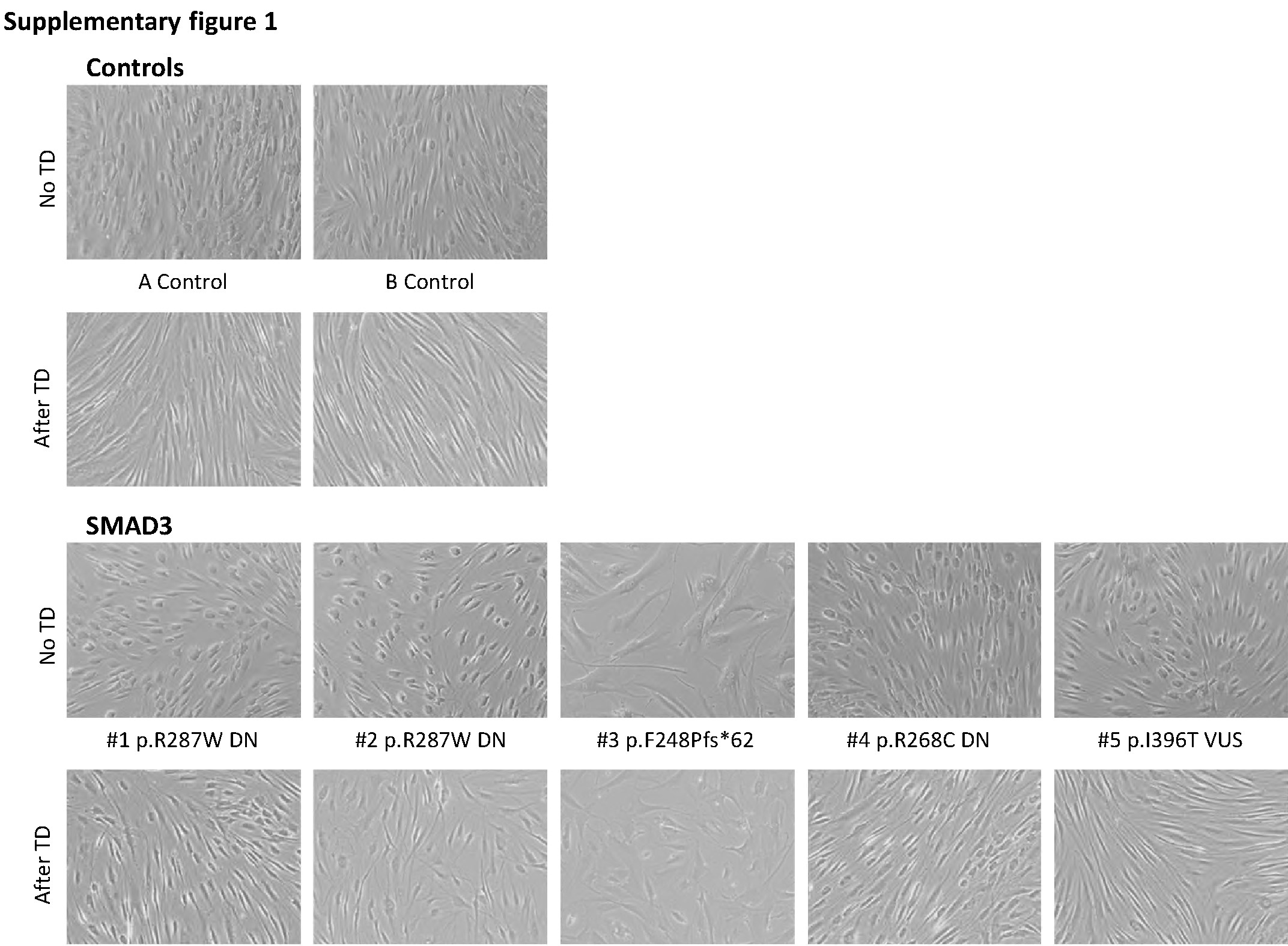

Supplement: Supplementary_Figure_1_ddae044 [file supplementary_figure_1_ddae044.jpeg]

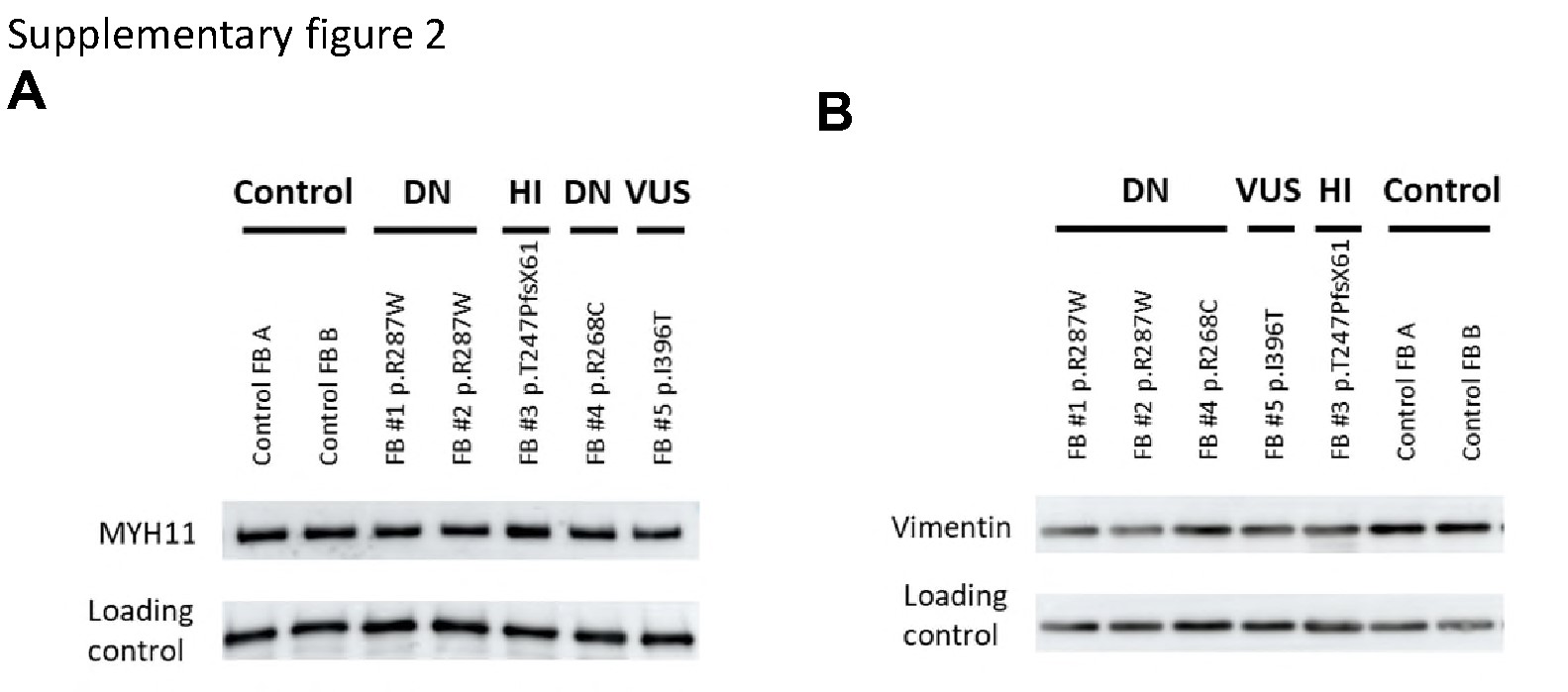

Supplement: Supplementary_Figure_2_ddae044 [file supplementary_figure_2_ddae044.jpeg]

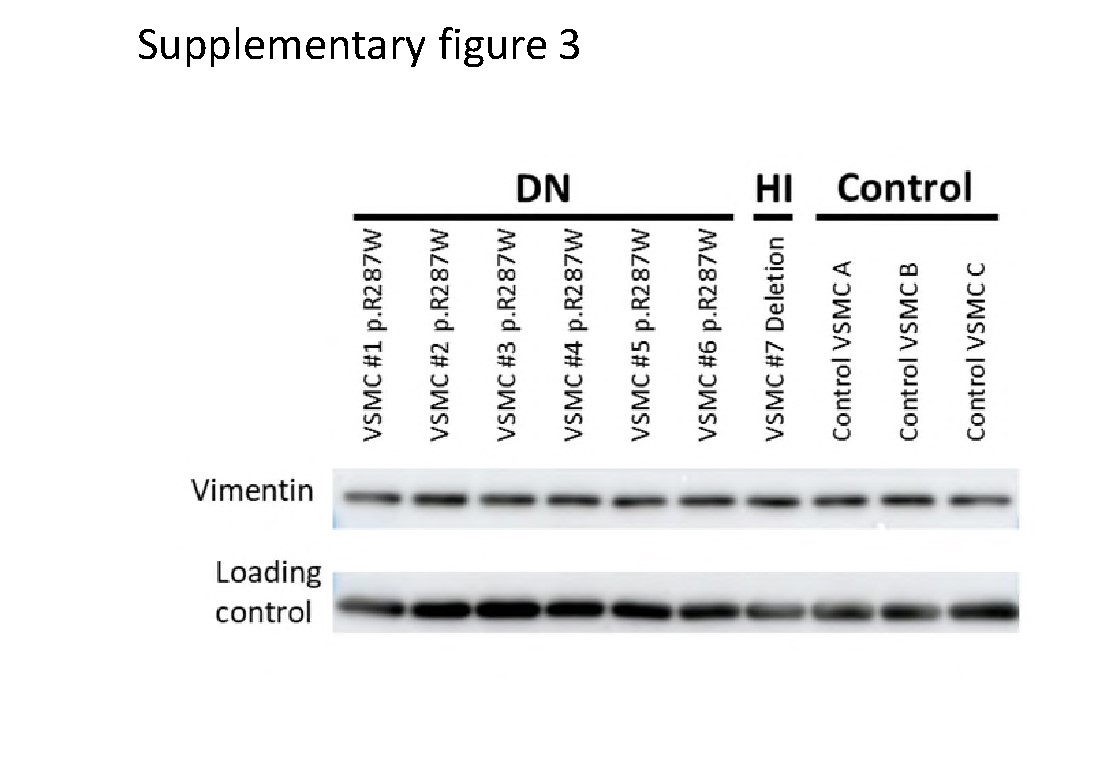

Supplement: Supplementary_Figure_3_ddae044 [file supplementary_figure_3_ddae044.jpeg]

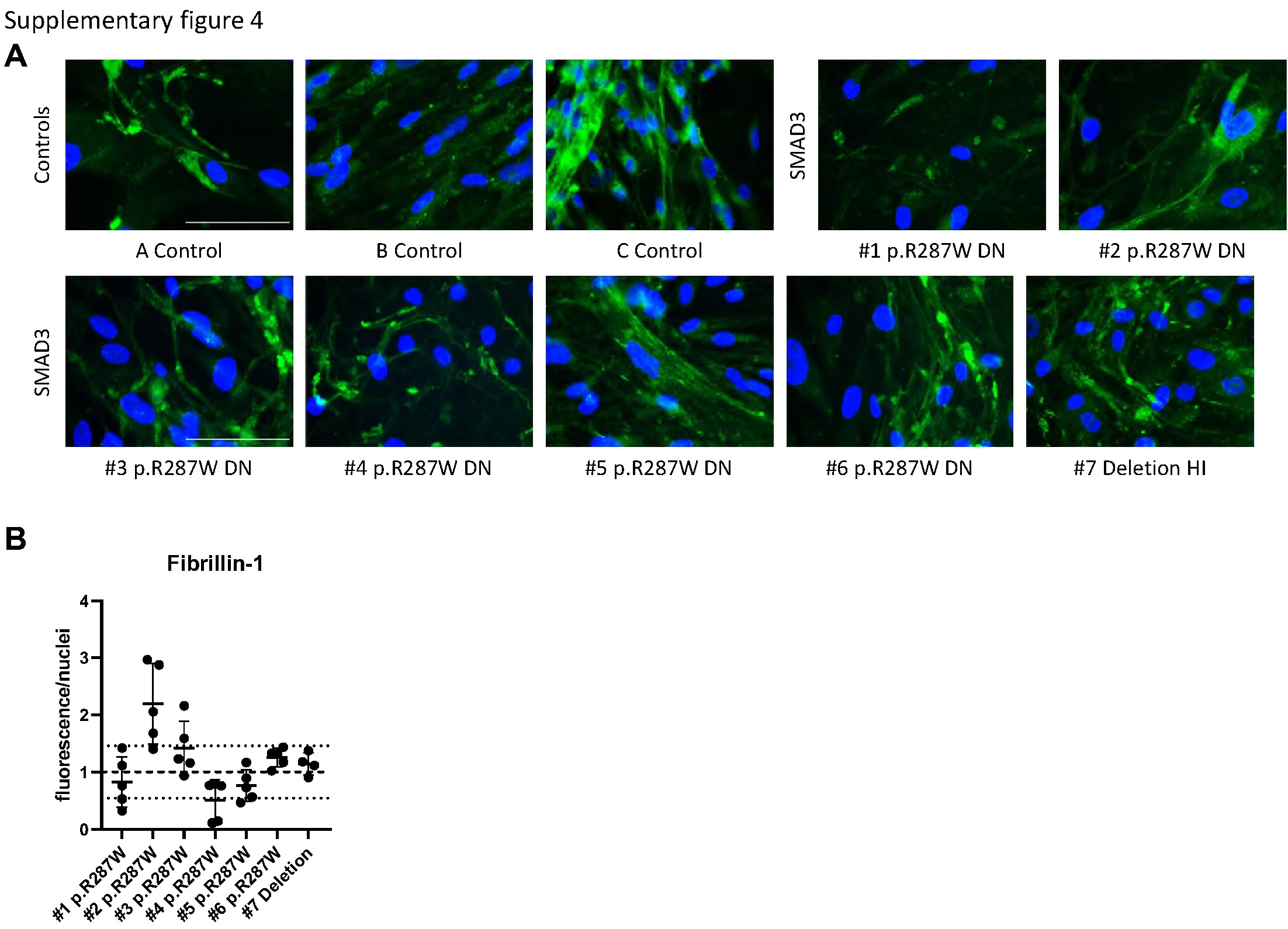

Supplement: Supplementary_Figure_4_ddae044 [file supplementary_figure_4_ddae044.jpeg]
