# Supplementary material for: Functional analysis of cell lines derived from SMAD3-related Loeys-Dietz syndrome patients provides insights into genotype-phenotype relation
Source: Hum Mol Genet. 2024 Mar 27;33(12):1090–104. doi: 10.1093/hmg/ddae044 (PMC11153339; doi:10.1093/hmg/ddae044)
Supplement: Supplementary_material_ddae044 [file supplementary_material_ddae044.pdf]

SUPPLEMENTARY MATERIAL

Supplementary table 1. Overview of identified pathogenic and likely pathogenic *SMAD3* variants

Location	Domain	Nucleotide change	RNA and/or protein change	Coding effect	MAF gnomAD	Pathogenic effect predicted (in silico)[†]	CADD score	Evidence[#ΔΥ*]	Classification	No. patients	Ref
Exon 1	MH1	c.76C>T	p.(Gln26*)	Nonsense	Absent	-	-	PVS1_very strong PM2_mod	Likely pathogenic	2	
Exon 2	MH1	c.304G>A	p.(Glu102Lys)	Missense	Absent	3/4	29.7	PM2_mod, PM1_sup, PP2_sup, PP3_sup, PP1_sup	Likely pathogenic	1	(56)
Intron 2	MH1	c.401-6G>A	p.(Val134Aspfs*33, r.400_401insACAG)	Frameshift	Absent	-	-	PVS1_very strong, PM2_mod	Likely pathogenic	3	(24, 57)
Exon 6	Linker	c.(658+1_659-1)_(871+1_872-1)del	p.(Asp220_lle290del)	In frame deletion	Absent	-	-	PVS1_strong, PM2_mod	Likely pathogenic	1	
Exon 6	MH2	c.716A>G	p.(Glu239Gly)	Missense	Absent	4/4	31.0	PS1, PM1_mod, PP2_sup, PM2_mod, PM5_mod, PP3_mod	Pathogenic	3	(57, 58)
Exon 6	MH2	c.741-742delAT	p.(Phe248Profs*62)	Frameshift	Absent	-	-	PVS1_very strong, PM2_mod, PP1_sup, PP4_sup	Pathogenic	4	(6)
Exon 6	MH2	c.802C>T	p.(Arg268Cys)	Missense	Absent	4/4	32.0	PS4_mod, PM1_mod, PM2_mod, PM5_mod, PP2_sup, PP3_sup	Likely pathogenic	2	(29)
Exon 6	MH2	c.859C>T	p.(Arg287Trp)	Missense	Absent	4/4	22.4	PM1_mod, PP2_sup, PM2_mod, PM5_mod, PP3_sup, PP1_sup, PP4_sup	Likely pathogenic	32	(6, 57, 59)
Exon 6	MH2	c.861delG	p.(Arg288Aspfs*53)	Frameshift	Absent	-	-	PVS1_very strong, PM2_mod, PP4_sup	Likely pathogenic	4	(24)
Exon 8	MH2	C.1045G>C	p.(Ala349Pro)	Missense	Absent	4/4	29.7	PM2_mod, PP2_sup, PP3_strong, PP4_sup	Likely pathogenic	3	(5)
Exon 8	MH2	c.1102C>T	p.(Arg368*)	Nonsense	Absent	-	-	PVS1_very strong, PM2_mod, PP4_sup, PP5_sup	Pathogenic	7	(60)
		Deletion from exon 2 onwards: Chr2.hg19: g.(67,408,242	p.(Ser70Aspfs*49)	Frameshift	Absent	-	-	PVS1_very strong, PM2_mod, PP1_sup, PP4_sup	Pathogenic	5	(30)

		)_(67,603,013)del									

NCBI Reference Sequence: NM_005902.4, NC_000015.10. gnomAD, Genome Aggregation Database v2.1.1; MAF, minor allele frequency; CADD, Combined Annotation Dependent Depletion; Ref, reference

[ǂ] Based on MutationTaster, PolyPhen2, SIFT, align GVGD

[#] PVS1: Probability of loss of function intolerance (pLI) score for SMAD3 is 0.8; loss of function observed/expected upper bound fraction (LOEUF) score for SMAD3 is 0.4. Interpretation of PVS1 is based on the recommendations of Abou Tayoun et al. (61).

[Δ] PS4 is given after calculating BayesianOR according to the recommendation of Kyung Cho et al (62). Odds ratio are > 5.0 and confidence interval does not include 1.0.

[ϒ] Specification of PP2; The missense constraint score (Z score ≥3.09) from gnomAD is used (63, 64). Z-score for SMAD3 is 3.48 (https://gnomad.broadinstitute.org)

[*] PP4 is given when the patients phenotype includes aneurysms AND osteoarthritis

*Suspected based on prediction models in Alamut.

Supplementary table 2. Available *SMAD3* patient and control cell lines

***SMAD3* variant**	**VSMCs**	**Fibroblasts**
p.(Arg287Trp)	6 patients (VSMC #1-#6)	2 patients (FB #1 and #2)
p.(Phe248Profs*62)		1 patient (FB #3)
p.(Arg268Cys)		1 patient (FB #4)
p.(Ile396Thr) (VUS)		1 patient (FB #5)
Deletion from SMAD3 exon 2 onwards	1 patient (VSMC #7)	
Control	3 cell lines (VSMC A, B, C)	2 cell lines (FB A and B)

Supplementary table 3. Patient characteristics

Cell line	FB #1	FB #2	FB #3	FB #4	FB #5	VSMC #1	VSMC #2	VSMC #3	VSMC #4	VSMC #5	VSMC #6	VSMC #7
**cDNA change**	c.859C>T	c.859C>T	c.741-742delAT	c.802C>T	c.1187T>C	c.859C>T	c.859C>T	c.859C>T	c.859C>T	c.859C>T	c.859C>T	Deletion 1 allele
**Protein change**	p.(Arg287Trp)	p.(Arg287Trp)	p.(Phe248Profs*62)	p.(Arg268Cys)	p.(Ile396Thr)	p.(Arg287Trp)	p.(Arg287Trp)	p.(Arg287Trp)	p.(Arg287Trp)	p.(Arg287Trp)	p.(Arg287Trp)	p.(Ser70Aspfs*49)
**Sex**	M	M	M	F	M	F	M	M	M	M	M	M
**Predicted effect**	DN	DN	HI	DN	VUS	DN	DN	DN	DN	DN	DN	HI
**Age at diagnosis (years)**	30	11	42	55	55	41	20	18	31	63	22	19
**Age at surgery/ biopsy (years)**	33	32	43	56	55	41	20	18	31	63	22	25
**Thoracic aortic aneurysm**	+	+	+	-	-	+	+	+	+	+	+	+
**Aortic diameter (mm) at surgery**	NA	NA	NA	NA	NA	39	40	46	54	43	50	45
**Aortic Z-score**	NA	NA	NA	NA	NA	2.69	3.07	4.96	7.69	2.6	6.5	3.91
**Arterial aneurysm elsewhere**	+	-	-	+	+	+	-	-	+	+	-	-
**Arterial tortuosity**	+ Cerebral arteries	-	-	-		-	+ Aortic root	-	-	-	-	-
**Total score other features[1]**	1	3	0	1	5	1	2	1	1	2	0	0

FB, fibroblast; VSMC, vascular smooth muscle cell; M, male; F, female; DN, dominant negative; HI, haploinsufficient; VUS, variant of unknown significance; NA, not available. Reference sequence: NM_005902.4.

[1] Other features (n=8) include: osteoarthritis of ≥1 joint, intervertebral disc degeneration, osteochondritis dissecans, scoliosis, pectus deformity, hypertelorism and/ or deep-set eyes, abnormal palate and/ or uvula, velvety skin and/ or abnormal striae

Supplementary table 4. Primary antibodies used for Western blotting

Primary antibody	Predicted kDA	Dilution	Manufacturer	Catalog number
Rabbit α-SM22 IgG	23	1:2000	Abcam	Ab14106
Mouse α-SMA IgG2a	42	1:10.000	Abcam	Ab7817
Rabbit α-MYH11 IgG	227	1:1000	Abcam	Ab53219
Rabbit α-SMAD3 IgG	48	1:1000	Abcam	Ab28379
Rabbit α-pSMAD3 IgG	48	1:1000	Abcam	Ab52903
Rabbit α-Smad2 IgG	60	1:1000	Cell Signaling	5339S
Rabbit α-pSmad2 IgG	60	1:400	Merck Millipore	04-953
Rabbit α-HSP60 IgG	61	1:10.000	GeneTex	GTX110089
Mouse α-GAPDH IgG1	40	1:5000	Abcam	Ab8245
Rabbit α-Vimentin	54	1:1000	Abcam	Ab92547

Supplementary table 5. ECM antibodies

**Primary antibody**	**Dilution**	**Manufacturer**	**Catalog number**
Rabbit α-Fibronectin IgG	1:80	Abcam	Ab2033
Rabbit α-mouse fibrillin-1	1:1000	Generated in Reinhardt lab	-
Rabbit α-mouse fibulin-4	1:500	Generated in Reinhardt lab	-
Rabbit α-mouse fibulin-5	1:500	Generated in Reinhardt lab	-

LEGENDS TO SUPPLEMENTARY FIGURES

Supplementary figure 1. Morphology of fibroblasts

Caption: Morphology before and after transdifferentiation for 14 days.

Alt text: Decorative bright field microscopy pictures of fibroblasts before and after TGF-β stimulation.

Supplementary figure 2. MYH11 and Vimentin on transdifferentiated fibroblasts

A) Caption: Western blot detecting MYH11 in transdifferentiated fibroblasts. B-catenin levels serve as a loading control.

Alt text: Decorative Western blots of the MYH11 expression.

B) Caption: Western blot detecting Vimentin in transdifferentiated fibroblasts. GAPDH levels serve as a loading control.

Alt text: Decorative Western blots of the Vimentin expression.

Supplementary figure 3: Vimentin VSMCs

Caption: Western blot detecting Vimentin in VSMCs. GAPDH levels serve as a loading control.

Alt text: Decorative Western blots of the Vimentin expression.

Supplementary figure 4: ECM proteins in VMSC

A) Caption: Immunofluorescent images show Fibrillin-1 staining after 14 days of culture. Scale bar represents 100 µm.

Alt text: Decorative fluorescence microscopy pictures of combined staining for Fibrillin-1 and DAPI.

B) Caption: Quantification of Fibrillin-1 staining after 14 days of culture.

Alt text: Decorative individual value plot with mean and standard deviation of fibrillin-1 immunofluorescence.
